# Supplementary material for: Tracing the electron transport behavior in quantum-dot light-emitting diodes via single photon counting technique
Source: Nat Commun. 2024 Sep 17;15:8150. doi: 10.1038/s41467-024-52521-0 (PMC11408697; doi:10.1038/s41467-024-52521-0)
Supplement: Supplementary file 1 — Supplementary Information [file 41467_2024_52521_MOESM1_ESM.pdf]

Supplementary Information for

Tracing the electron transport behavior in quantum-dot light-emitting diodes via single photon counting technique

Qiang Su[1,2], Zinan Chen[1], Shuming Chen[1,*]

[1]Department of Electrical and Electronic Engineering, Southern University of Science and Technology, Shenzhen 518055, P. R. China

[2]School of Physical Sciences, Great Bay University, Dongguan 523000, P. R. China

* Corresponding author: Shuming Chen (chen.sm@sustech.edu.cn)

Supplementary Note 1. Simultaneous electroluminescence-photoluminescence (EL-PL) measurement.

The schematic configuration of the home-built EL-PL co-measurement system is shown in Figure 1e in the main text. In the experiment, we simultaneously obtained a mixed signal consisting of EL and PL. Specifically, the PL was excited in an alternating current mode, while the EL was set to direct current mode. Subsequently, by employing a lock-in amplifier, the alternating current PL signal was successfully extracted from the mixed signal, and the EL signal was obtained through the direct current coupling mode of an oscilloscope. This test enables in-situ monitoring of the optically pumped information of quantum dots (QDs) while operating quantum-dot light-emitting diodes (QLEDs) under electrical pumping conditions. This allows us to differentiate the impact of γ and η_r on the external quantum efficiency (EQE).

To obtain accurate results, several considerations need to be taken into account:

(1) Select an appropriate excitation light to avoid exciting other functional layers. In this case, a 532 nm laser was used.

(2) In order not to collect the excitation and the TFB signals, a long-pass filter was added in front of the detector, so that only the red emission from the QDs can be detected.

(3) The power of excitation laser needs to be maintained at a low level to prevent Auger quenching and aging of QDs. Therefore, in this study, the laser power was set to 1.0–2.5 mW cm^{-2}. Supplementary Figure 1 illustrates the variation of PL intensity of a red QLED during a continuous laser excitation of 3 hours with a power of 1.0–2.5 mW cm^{-2}. It can be observed that there is no deterioration in the optical performance of the QDs within the 3-hour period, thereby confirming the rationality of the excitation intensity set in our experiment.

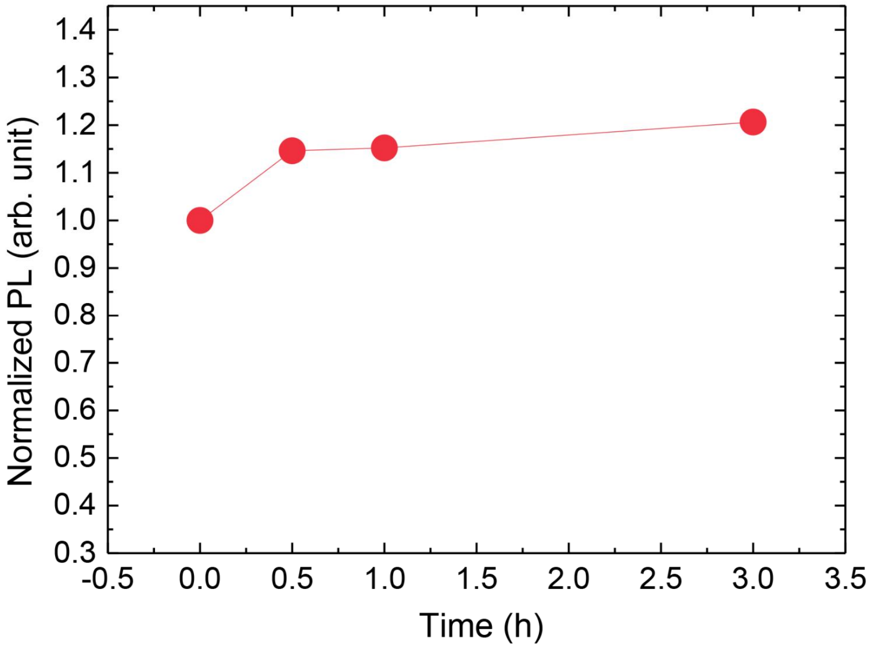

Supplementary Figure 1. PL intensity of a red QLED under continuous excitation. There is no deterioration in the PL of the QDs within the 3-hour period.

(4) The EL signal acquisition method in this experiment is as follows: Under the applied target voltage, the direct current EL signals obtained from the oscilloscope is divided by the current of the device at that driving voltage. To ensure accuracy, the EL data in Figure 1b was collected in two segments. The EL data ranging from 1.5 V to 3.5 V was obtained from J-V-L characterization, while the EL data above 3.5 V was obtained from the EL-PL co-measurement.

In addition, in Figure 1b, the PL signal remains at its maximum value within the voltage range of 1.5–2.2 V, while it exhibits significant attenuation at 0 V or −1.5 V. This phenomenon is believed to be caused by the influence of the built-in electric field on the photogenerated excitons. As illustrated in Supplementary Figure 2, at $V = 0$ V, the built-in electric field tends to push the photogenerated excitons out of the recombination zone, causing their dissociation; at $V = -1.5$ V, the external electric field is consistent with the built-in electric field, leading to more pronounced exciton dissociation; conversely, at $V = 1.5$ V, the external electric field opposes the

built-in electric field, inhibiting the dissociation of photogenerated excitons and favoring their localization within the recombination zone. In summary, the interplay between the electric field and exciton dissociation/localization results in variations in the intensity of the PL signal. The normalization of the PL signal in this study is based on the maximum point.

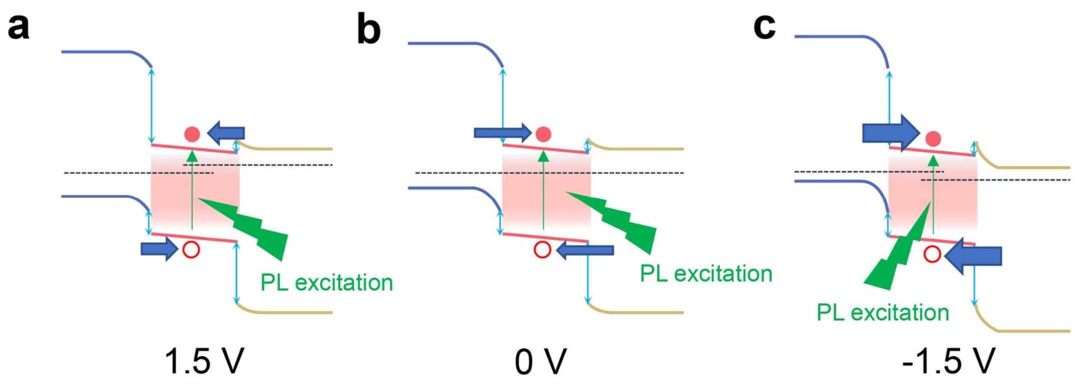

Supplementary Figure 2. The effect of electric field on photogenerated excitons. In a state of thermal equilibrium, an internal built-in electric field is established within the device. The green arrows in the diagram represent the generation of photogenerated excitons under optical excitation. The blue arrows indicate the direction of the effect of external electric field on the photogenerated excitons.

Supplementary Note 2. The extraction of the γ

From t*he EQE de*finition:

$$EQE(V) = \gamma(V) * \eta_r(V) * \eta_c \quad (1)$$

$\gamma(V)$ is equal to:

$$\gamma(V) = \frac{EQE(V)}{\eta_c * \eta_r(V)} \quad (2)$$

The EL and the PL of QLED were simultaneously measured using our home-built EL-PL co-measurement system. We made some reasonable replacements in the above equation: the EQE is replaced with $EQE_{peak} * EL_{normalized}(V)$; η_r is replaced with $\eta_r * PL_{normalized}(V)$, and η_c is a constant. Thus, $\gamma(V)$ is obtained from the following equation,

$$\gamma(V) = \frac{EQE_{peak} * EL_{normalized}(V)}{\eta_c * \eta_r * PL_{normalized}(V)} \quad (3)$$

The $EL_{normalized}(V)$, and $PL_{normalized}(V)$ were obtained by measuring the EL-V and PL-V characteristics simultaneously. The $EL_{normalized}(V)$ is corrected based on the EL signal and the driving current, so that the obtained $EL_{normalized}(V)$ actually reflects the normalized EQE of the devices. The resulting $EQE_{peak} * EL_{normalized}(V)$ is almost the same as EQE (V) of the devices, except that it also reflects the perturbation of PL excitation on QDs. In addition, because the PL is only affected by η_r, the $\eta_r * PL_{normalized}(V)$ therefore reflects the $\eta_r(V)$.

Supplementary Note 3. Tracing the leakage electrons in QLEDs via single photon counting (SPC) technique.

The schematic measurement setup is shown in Figure 3a in the main text. The SPC technique utilizes the inherent discrete nature of photon detector output signals under weak light illumination. By employing pulse discrimination and digital counting techniques, it allows for the identification and extraction of extremely faint light signals. In this study, the SPC integrated within the Edinburgh FS5 system was utilized, which is commonly employed for fluorescence lifetime measurements. We employed this system to detect the weak light signals generated within the QLED due to electron leakage. The setup also incorporates a grating monochromator that enables the selective detection of the photons at a specific wavelength.

During the testing process, the QLED sample was placed at the receiving end of the single photon counter, and the testing area was sealed to isolate ambient light. Subsequently, by driving the QLED through a source meter, the number of photons was recorded for each spectrum at different driving voltages.

Other details:

(1) The grating monochromator integrated in the single photon counter enables the selective detection of the photons at a specific wavelength. The split signal goes directly to the detector without additional amplification.

(2) The detector in the single photon counter is calibrated with a broad-spectrum standard light source, ensuring an accurate and error-free response at visible wavelength.

(3) The kinetic scan mode with a corresponding bandwidth was used during the test. The photon counts for each data point were averaged over a collection time of 30 s. During the testing, the emission of the QLED was very stable.

(4) The ratio in Figures 3e, 3f and 3g is mainly calculated based on the EL results detected by single photon counter.

(5) The device position was fixed during testing.

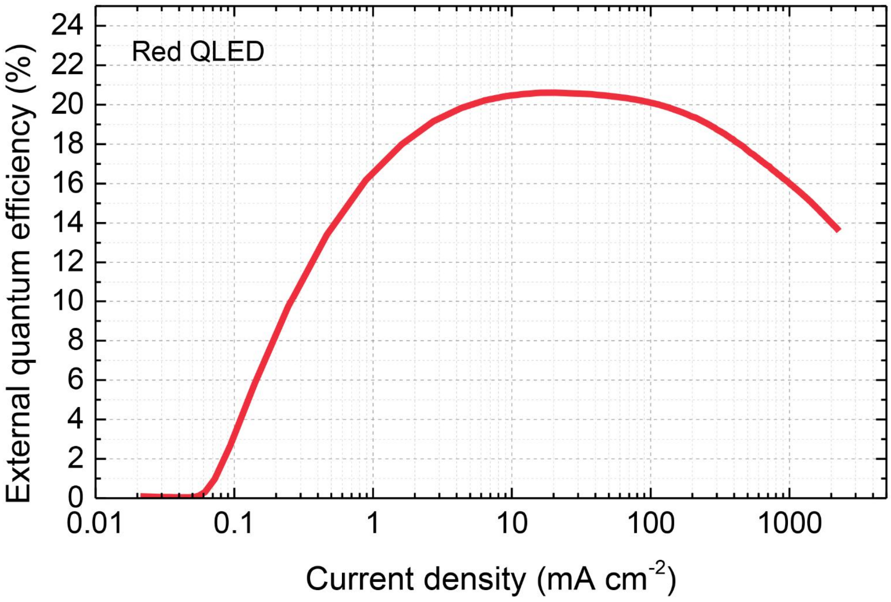

Supplementary Figure 3. External quantum efficiency-current density (EQE-J) characteristics of a red QLED. Not all electrons are confined within the QDs and subsequently converted into photons, especially when the devices are operated at a small J or a large J. The results are the EQE is very low at small J and the QLED has a significant efficiency roll-off at large J.

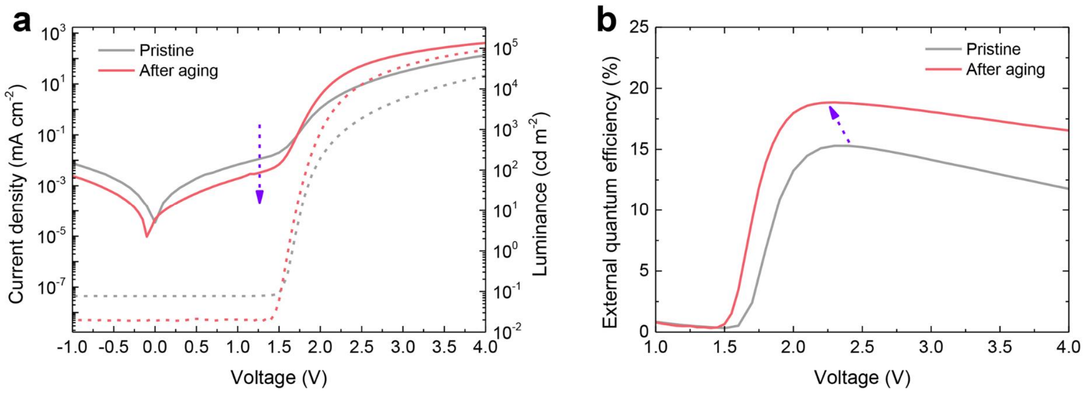

Supplementary Figure 4. Positive aging in red QLEDs. a Current density-voltage-luminance (*J-V-L*) and **b** EQE-*J* characteristics of a red QLED before and after positive aging. To exclude the effect of positive aging [1, 2] on device characteristics, the devices were encapsulated and shelf-stored in N_2 glove box for several days, so that the positive aging process is fully completed. All devices under test exhibit a stable efficiency and uniform emission, as shown in Figure 1f in the main text, confirming that the positive aging effect has been ruled out.

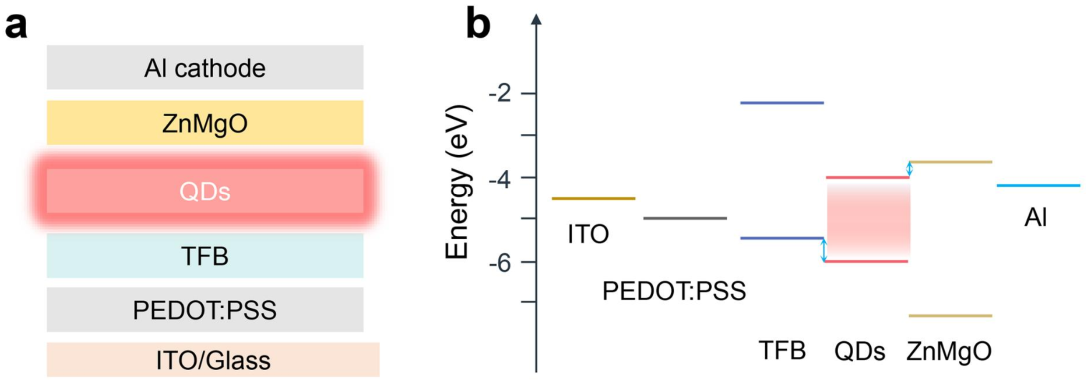

Supplementary Figure 5. The typical red QLED. a Device structure and **b** energy band diagram of the red QLED. The blue and green QLED used in the manuscript have the same structure as red QLED.

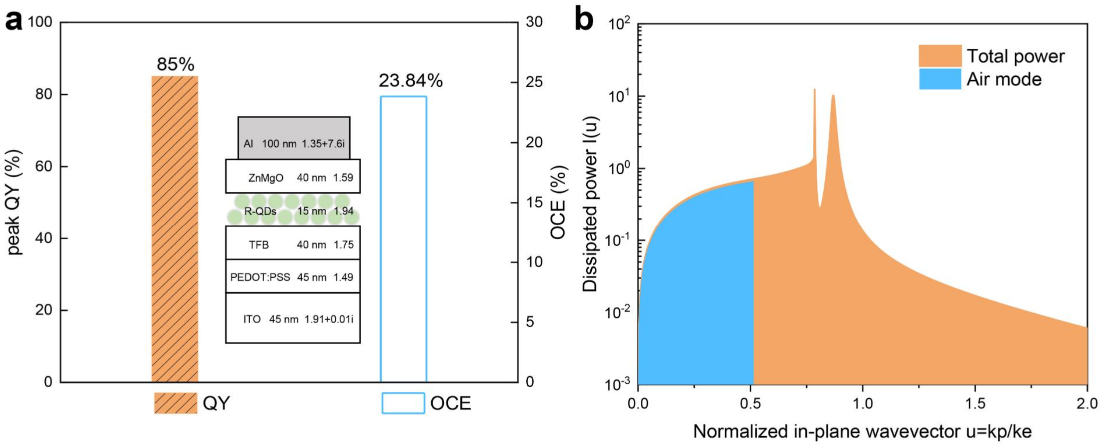

Supplementary Figure 6. Light outcoupling efficiency. The outcoupling efficiency (OCE) of a red QLED with a structure of glass/ITO (45 nm)/PEDOT:PSS (45 nm)/TFB (40 nm)/R-QDs (15 nm)/ZnMgO (40 nm)/Al (100 nm). It can be seen that the OCE in red QLED is 23.84%. Therefore, we assume that 25% of OCE is reasonable. Optical properties of the functional layer are referenced form our previous report [3].

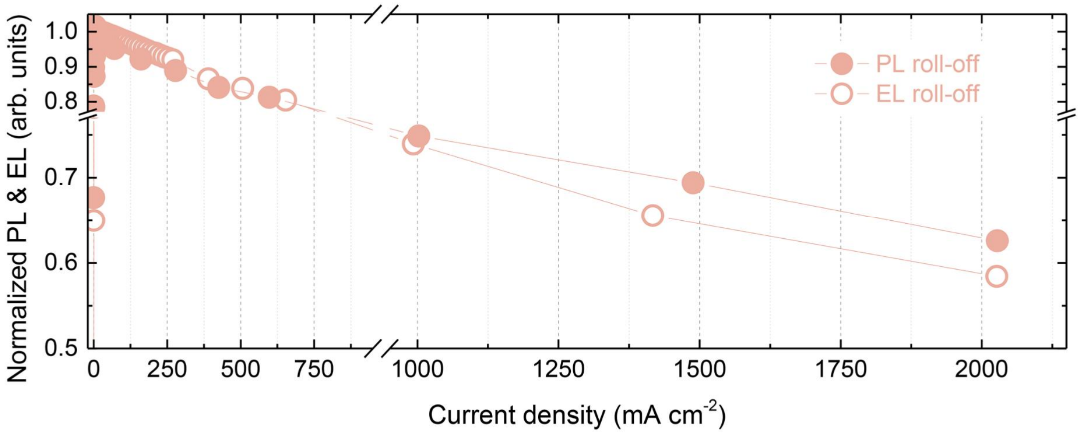

Supplementary Figure 7. The simultaneous PL-*V* and EL-*V* characteristics of a red QLED at the current density scale. At a high current density J of 993 mA cm^{-2} (corresponding to a large voltage of 6 V, see Figure 1d), the EL decreases more rapidly than the PL, suggesting a decrease in γ with increasing voltage.

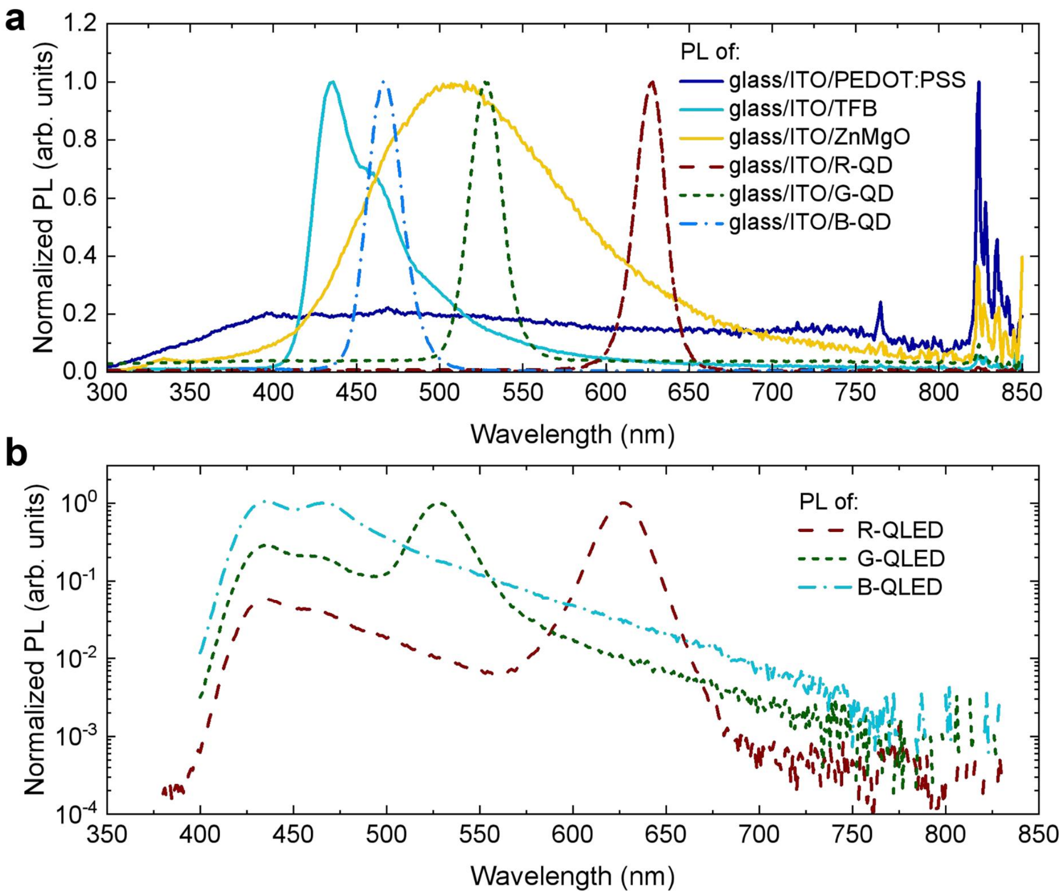

Supplementary Figure 8. Confirmation of TFB fluorescent emission localization. **a** The PL emission spectra of all functional layers (PEDOT:PSS, TFB, ZnMgO) and the red, green, and blue QDs. **b** The PL emission spectra of red, green, blue QLEDs. Based on the results of PL, it can be concluded that the emission in the range of 490–500 nm originates only from TFB. Firstly, the luminescence of QDs is highly pure. Secondly, PEDOT:PSS does not exhibit fluorescence emission, while the emission of ZnMgO mainly occurs between 500–550 nm [2, 4]. Although the TFB has multiple PL emission peaks, its EL resulted from the leakage electrons only exhibits the 490-500 nm emission. Considering the disorder of TFB, its LUMO actually consists of multiple energy levels. Most of the leakage electrons can only overflow to the lower LUMO levels of TFB, thus resulting in a long wavelength emission of 490-500 nm. In other words, the energy of electrical excitation caused by leakage electrons is much lower than that of optical excitation, thus resulting in a longer wavelength EL emission.

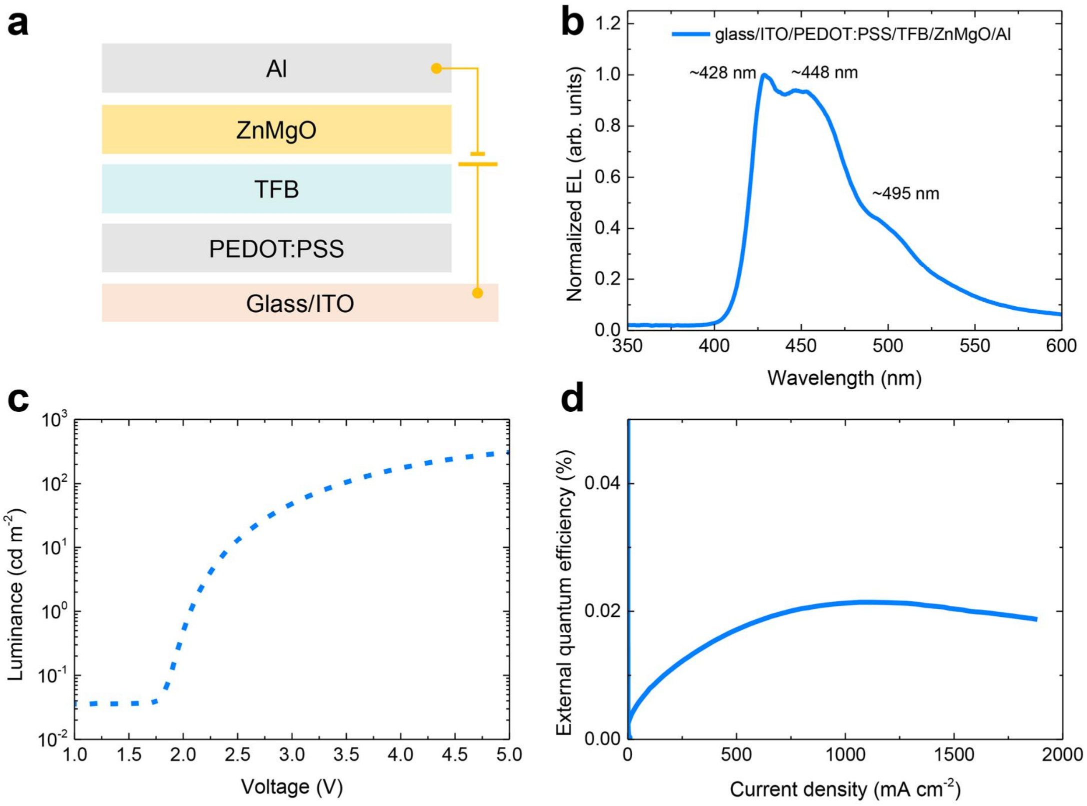

Supplementary Figure 9. Confirmation of TFB fluorescent emission localization. To further verify the fluorescent emission of TFB, EL device with TFB as the emissive unit was fabricated. **a** Device structure ： glass/ITO/PEDOT:PSS/TFB/ZnMgO/Al. **b** The EL spectrum of device**.** Three distinct emission peaks are observed for TFB at approximately 430 nm, 450 nm, and around 495 nm, corresponding to the PL spectra. Additionally, the detected TFB emission resulting from electron leakage only exhibits in the range of 490-500 nm, indicating insufficient EL excitation of TFB [5]. The device exhibits low **c** luminance and **d** EQE, partly due to inefficient electron injection in the device and also because of the low quantum yield of TFB.

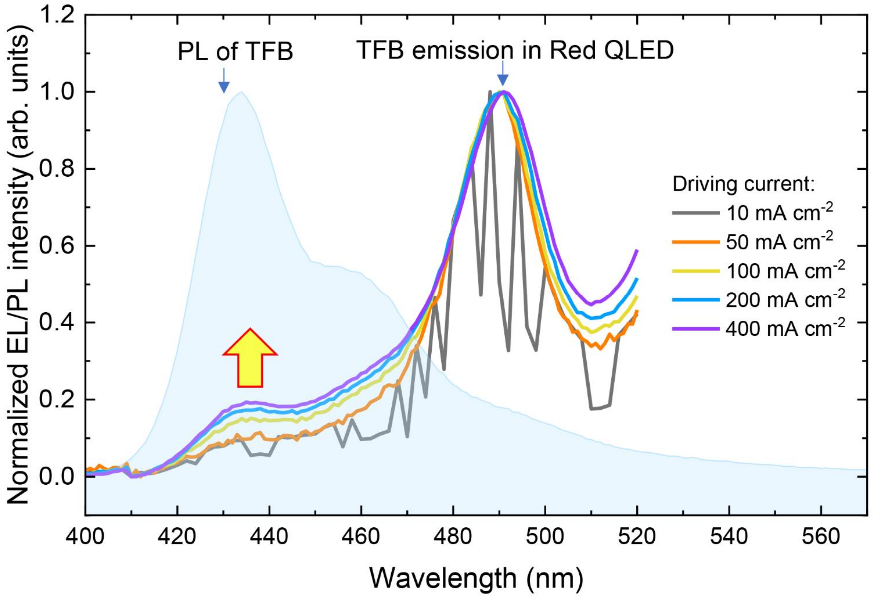

Supplementary Figure 10. Confirmation of TFB fluorescent emission localization. The lack of TFB emission within the 420-450 nm range indicates the insufficient EL excitation of TFB. To further confirm this point, we examined the TFB emission in the red QLED at different driving currents. The EL spectra showed a gradual increase in the 420-450 nm region as the driving current was increased, proving that the increase in the number of leakage electrons could more fully excite the fluorescence emission of TFB. However, it should be noted that since the percentage of leakage electrons in the device is small, the TFB emission is still mainly concentrated around 490 nm.

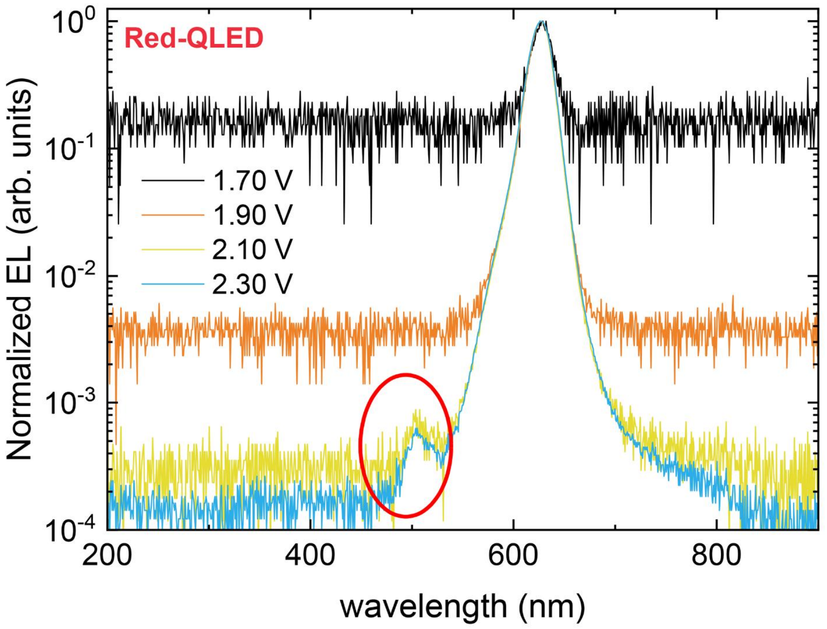

Supplementary Figure 11. Limitation of spectrometers in detecting weak light signals. In the experiment, a spectrometer was employed to acquire the spectra of a red QLED. Even with significantly increased integration time, only the emission information of TFB at 490-500 nm could be observed at drive voltages of 2.1 V and above. This finding not only indicates a previous misjudgment of TFB emission but also highlights the notable advantage of SPC in weak light detection.

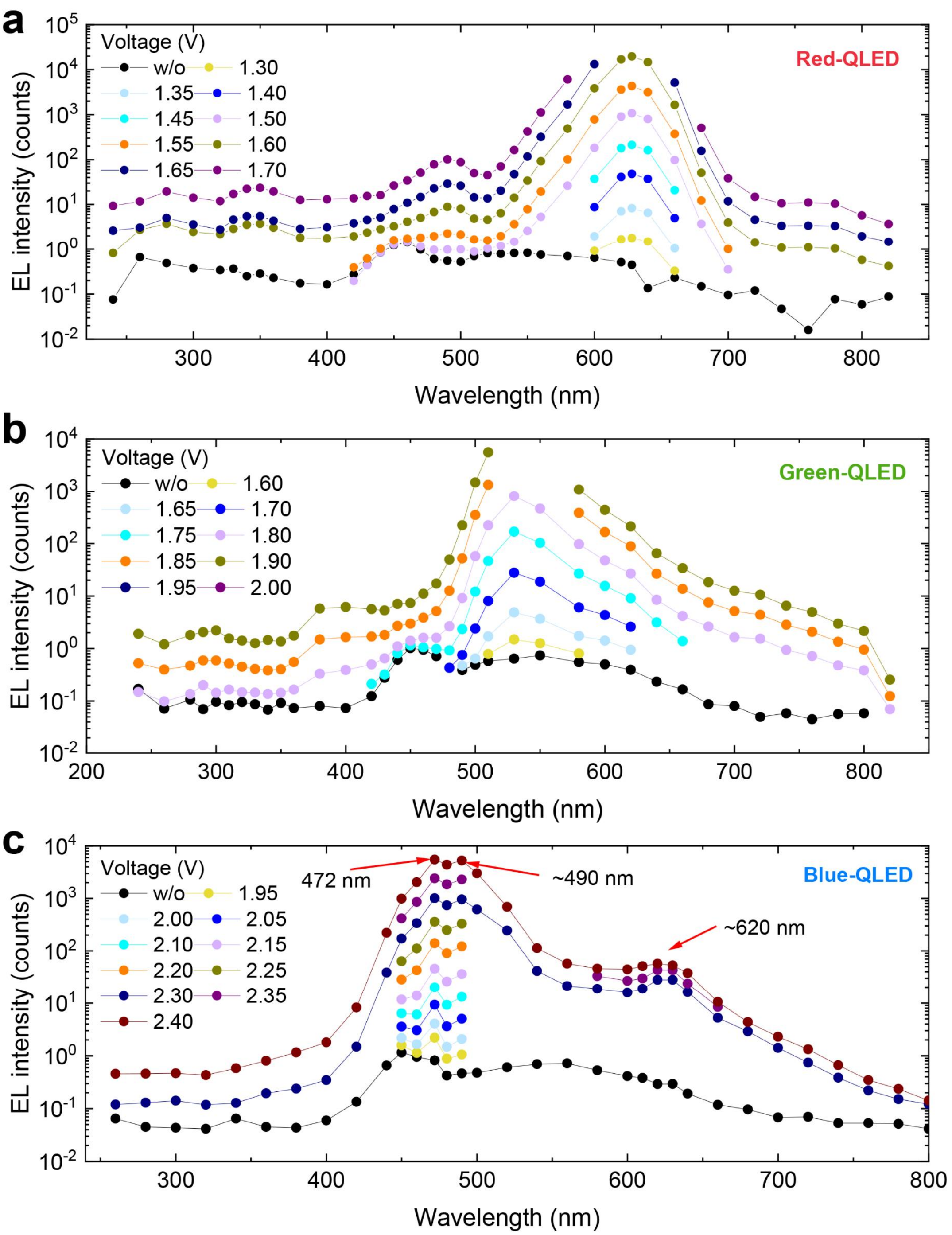

Supplementary Figure 12. QD emission and TFB emission in the red, green and blue devices. The emission spectra of **a** red, **b** green, and **c** blue QLEDs were acquired by utilizing SPC technique. On one hand, the emission of TFB (at 490 nm) is observed in all QLEDs and appears near the turn-on voltage. The information regarding green QLED is unclear due to the overlapping emission of the green QDs themselves with the TFB emission. However, based on the results from red and blue

ones, it can be inferred that there is electron leakage towards TFB in green QLED. On the other hand, emission information at 620 nm, indicating interface recombination, is observed specifically in blue QLED. At the current detection level, this information is only observed in blue QLED, but it cannot be concluded that the electron leakage channel does not exist in red and green QLEDs. Furthermore, under higher voltage driving conditions, electrons are more prone to overflowing into the TFB. Thus, under increased voltage driving (e.g., from V_T to 8.0 V), the emission from TFB persists alongside QD emission.

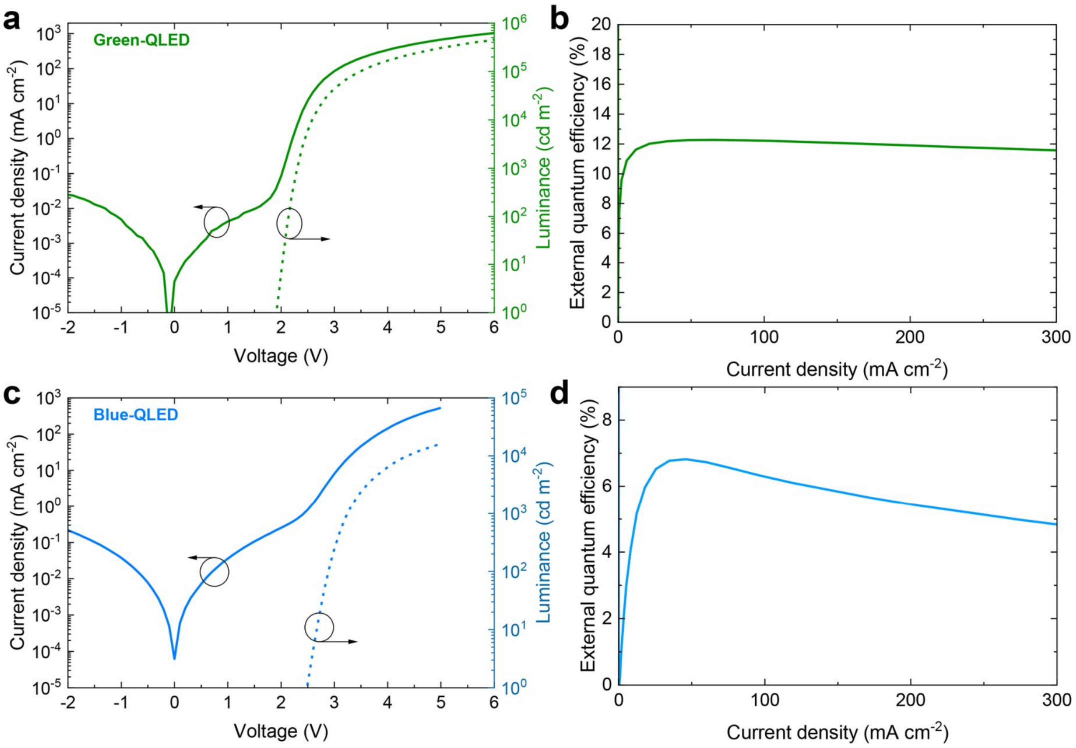

Supplementary Figure 13. The corresponding performance of green and blue QLEDs. The **ac** *J-V-L* and **bd** EQE-*J* characteristics of the corresponding **ab** green and **cd** blue QLEDs in Supplementary Figure 12.

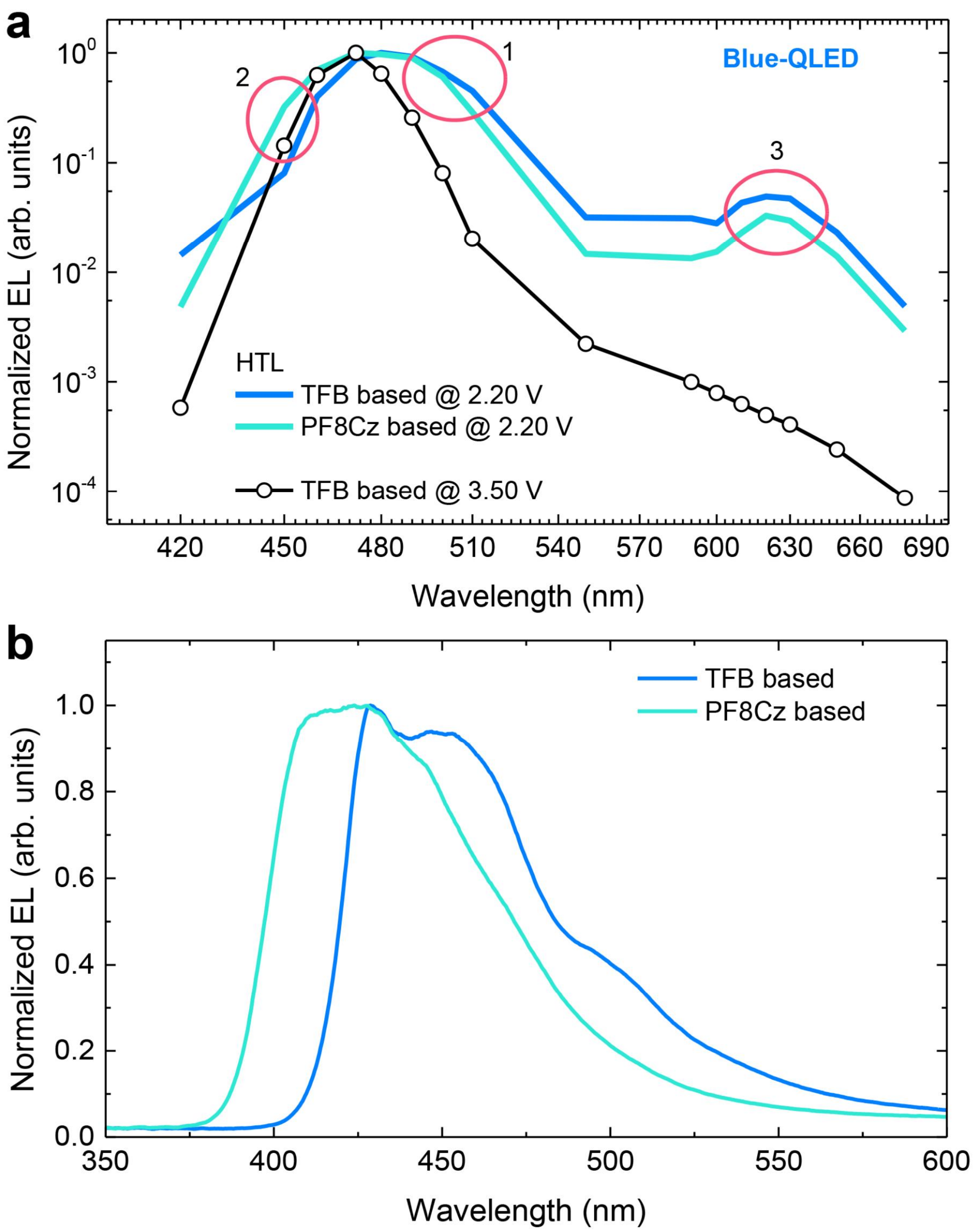

Supplementary Figure 14. Confirmation of the interfacial recombination. To further demonstrate the luminescent characteristics at 620 nm, we prepared a blue QLED with PF8Cz as the hole transport layer (HTL), as well as an EL device with PF8Cz as the emissive unit. The respective device structures were as follows:

glass/ITO/PEDOT:PSS/TFB (PF8Cz)/blue QDs/ZnMgO/Al,

glass/ITO/PEDOT:PSS/TFB (PF8Cz)/ZnMgO/Al.

a The spectra of blue QLEDs with different HTLs were obtained using SPC under

different driving voltages. Firstly, it can be observed that the device based on PF8Cz exhibits the same emission peak at 620 nm as the device based on TFB. Considering the similarity between the highest occupied molecular orbital (HOMO) levels of PF8Cz and TFB [6], the similarity in interface recombination energy is reasonable. This result further indicates that the 620 nm emission originates from the fluorescence emission resulting from the electron leakage at the HTL/QDs interface. **b** The spectra of EL devices with TFB and PF8Cz. It can be observed that the PF8Cz-based device exhibits a blue shift in the emission spectrum. This is attributed to the higher-lying lowest unoccupied molecular orbital (LUMO) level of PF8Cz compared to TFB, with an approximate upward shift of 0.2 eV [6]. Consequently, the bandgap energy of PF8Cz is larger, resulting in a blue shift in the emission spectrum. The blue shifts observed at marks 1 and 2 in **a** are a direct consequence of this phenomenon.

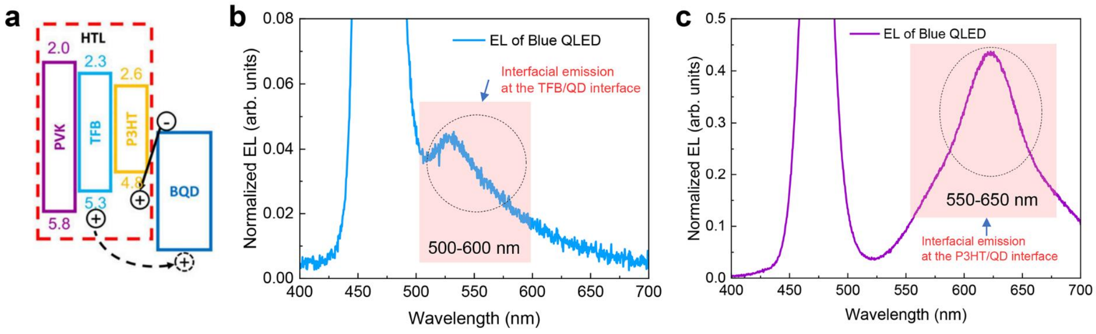

Supplementary Figure 15. Confirmation of the interfacial recombination. To further demonstrate the interfacial recombination, we reprinted the interfacial emission results from our previous work [7]. In principle, QLEDs with different HTLs should exhibit different interfacial emission peaks if the QD is the same. After replacing TFB with P3HT, there is a significant difference in the peak position of the interfacial emission and the significant redshift of the emission peak, which results from the higher HOMO level of P3HT [P3HT @ (-4.8 eV) vs TFB @ (-5.3 eV)]. This proves that the HOMO change of HTL causes a change in the energy of the interfacial emission, further supporting the existence of interfacial recombination.

In addition, there is a significant difference in the peak position of the interfacial emission reported previously compared to that in this work, which is due to the different blue QDs that we used.

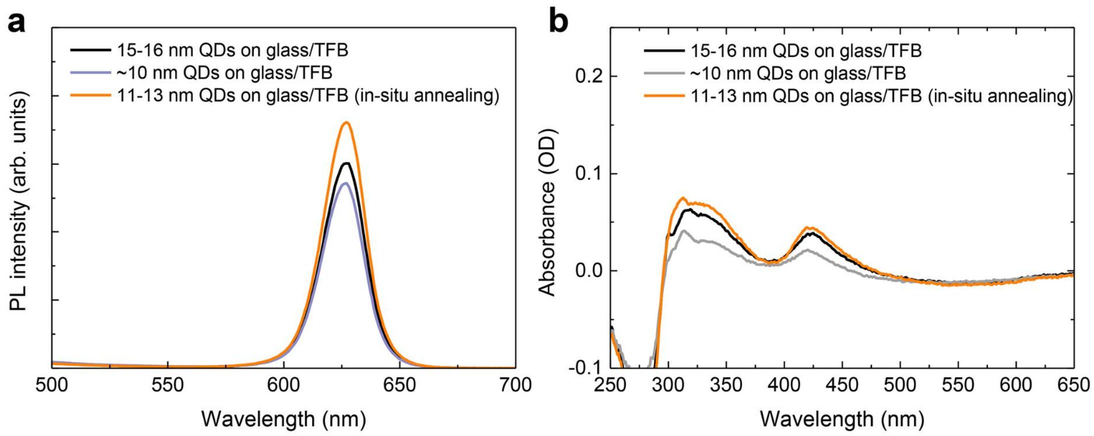

Supplementary Figure 16. Improving the compactness of the QD layers. To obtain a close-packed QD film, the QD solution was pre-heated (45 °C) prior to spin-coating, so as to improve their dispersion. Moreover, an in-situ annealing method (keep the substrate at 60 °C) was employed during the spin-coating process. Comparison of **a** PL intensity and **b** absorbance between conventionally spin-coated QD films and in-situ annealing spin-coated QD films. On one hand, the comparison between the black solid line and the gray solid line represents QD films with equal compactness but different thicknesses. It is evident that thicker QD films exhibit stronger PL intensity and higher optical density. On the other hand, the comparison between the orange solid line and the black solid line demonstrates that, as the thickness decreases, the in-situ annealing spin-coated QD film exhibits stronger PL intensity and absorbance. This indicates that the in-situ annealing spin-coating method significantly enhances the compactness of QD films.

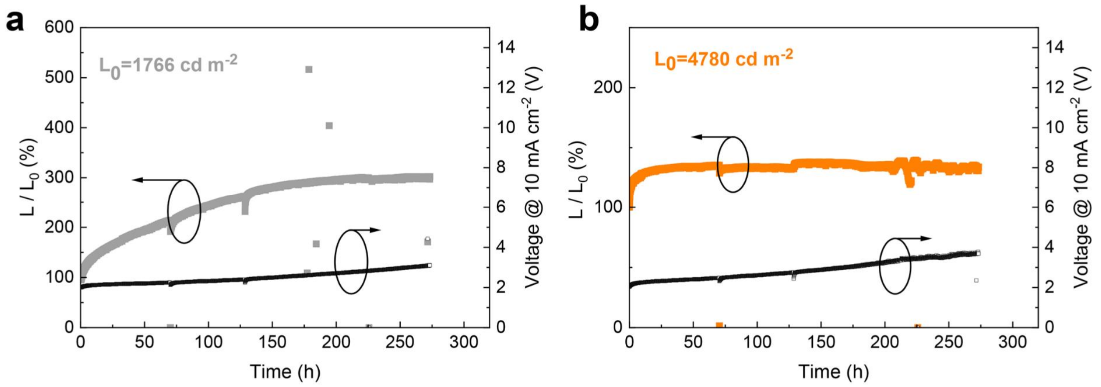

Supplementary Figure 17. Stability of QLED based on different QD films. The lifetime of red QLED with **a** conventionally spin-coated QD films and **b** in-situ annealing spin-coated QD films. It can be observed that the improved compactness of QD films enables higher brightness to be achieved at the same current. Meanwhile, the operational stability of QLEDs based new QD film remains unaffected, as evidenced by both devices exhibiting no brightness degradation after operating for over 250 hours.

Supplementary References

[1] Su, Q., Sun, Y. Z., Zhang, H. & Chen, S. M. Origin of positive aging in quantum-dot light-emitting diodes. *Adv. Sci.* **5**, 1800549 (2018).

[2] Chen, Z. N., Su, Q., Qin, Z. Y. & Chen, S. M. Effect and mechanism of encapsulation on aging characteristics of quantum-dot light-emitting diodes. *Nano Res*. **14**, 320–327 (2021).

[3] Li, H., Tian, F. & Chen, S. Approaching the theoretical efficiency limit of quantum-dot light-emitting diodes via synergistic optimization. *Nano Res.* **16**, 10156–10163 (2023)

[4] Kang, H. S., Kang, J. S., Kim, J. W. & Lee, S. Y. Annealing effect on the property of ultraviolet and green emissions of ZnO thin films. *J. Appl. Phys.* **95**, 1246–1250 (2004).

[5] Ye, Y. X. et al. Design of the hole-injection/hole-transport interfaces for stable quantum-dot light-emitting diodes. *J. Phys. Chem. Lett.* **11**, 4649–4654 (2020).

[6] Deng, Y. Z. et al. Solution-processed green and blue quantum-dot light-emitting diodes with eliminated charge leakage. *Nat. Photonics* **16**, 505–511 (2022).

[7] Huang, X. Y. et al. The influence of the hole transport layers on the performance of blue and color tunable quantum dot light-emitting diodes. *J. Soc. Inf. Display* **26**, 470–476 (2018).
